# Nuclear Projections in Neutrophils for Supporting the Diagnosis of Trisomy 13

**DOI:** 10.4274/tjh.2017.0227

**Published:** 2018-05-25

**Authors:** Şebnem Kader, Mehmet Mutlu, Filiz Aktürk Acar, Yakup Aslan, Erol Erduran

**Affiliations:** 1 Karadeniz Technical University Faculty of Medicine, Division of Neonatology, Trabzon, Turkey; 2 Karadeniz Technical University Faculty of Medicine, Division Pediatric Hematology, Trabzon, Turkey

**Keywords:** Trisomy 13, Blood smear, Neutrophilic nuclear projections

**To the Editor,**

Trisomy 13 is a rare genetic disorder characterized by severe multiple congenital anomalies. Structural anomalies of neutrophils may be supportive for the diagnosis of trisomy 13.

A newborn was born by vaginal delivery after 29 weeks of pregnancy. Physical examination revealed symmetric growth restriction, low-set hypoplastic ears, aplasia cutis congenita areata on the vertex, postaxial polydactyly of the foot, bilateral microphthalmia, an umbilical cord cyst, and heart murmurs. Echocardiography showed truncus arteriosus type I. Review of the peripheral blood smear revealed two or more small threadlike pedunculated projections attached to the surface of the nuclei in more than 60% of the neutrophils (Figure 1). The diagnosis of trisomy 13 was made by chromosomal analysis. The infant died at 2 days of life because of massive pulmonary hemorrhage.

The presence of threadlike pedunculated projections attached to the surface of the nuclei of neutrophils was described in trisomy of the D group of chromosomes (13, 14, and 15) and also in trisomy 18 [1,2]. Two or more nuclear projections detected in more than 15% of neutrophils may be highly suggestive of these trisomies [3]. We suggest that identification of characteristic structural anomalies of neutrophils on a blood smear may be used for supporting the diagnosis of these trisomies.

## Figures and Tables

**Figure 1:**
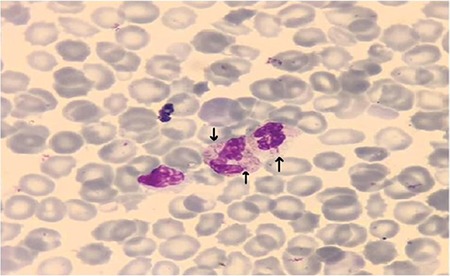
Peripheral blood smear showing threadlike pedunculated projections attached to the surface of the nuclei of neutrophils.
